# Pilot Scale Elimination of Phenolic Cellulase Inhibitors From Alkali Pretreated Wheat Straw for Improved Cellulolytic Digestibility to Fermentable Saccharides

**DOI:** 10.3389/fbioe.2021.658159

**Published:** 2021-03-12

**Authors:** Ikram ul Haq, Ali Nawaz, Badar Liaqat, Yesra Arshad, Xingli Fan, Meitao Sun, Xin Zhou, Yong Xu, Fatima Akram, Kankan Jiang

**Affiliations:** ^1^School of Basic Medical Sciences and Forensic Medicine, Hangzhou Medical College, Hangzhou, China; ^2^Institute of Industrial Biotechnology, Government College University, Lahore, Pakistan; ^3^Jiangsu Co-innovation Center of Efficient Processing and Utilization of Forest Resources, College of Chemical Engineering, Nanjing Forestry University, Nanjing, China

**Keywords:** green energy, green chemistry, phenols, bioenergy, lignocellulosic biomass, phenolic inhibitors

## Abstract

Depleting supplies of fossil fuel, regular price hikes of gasoline and environmental deterioration have necessitated the search for economic and eco-benign alternatives of gasoline like lignocellulosic biomass. However, pre-treatment of such biomass results in development of some phenolic compounds which later hinder the depolymerisation of biomass by cellulases and seriously affect the cost effectiveness of the process. Dephenolification of biomass hydrolysate is well cited in literature. However, elimination of phenolic compounds from pretreated solid biomass is not well studied. The present study was aimed to optimize dephenoliphication of wheat straw using various alkalis i.e., Ca(OH)_2_ and NH_3_; acids i.e., H_2_O_2_, H_2_SO_4_, and H_3_PO_4_; combinations of NH_3_+ H_3_PO_4_ and H_3_PO_4_+ H_2_O_2_ at pilot scale to increase enzymatic saccharification yield. Among all the pretreatment strategies used, maximum reduction in phenolic content was observed as 66 mg Gallic Acid Equivalent/gram Dry Weight (GAE/g DW), compared to control having 210 mg GAE/g DW using 5% (v/v) combination of NH_3_+H_3_PO_4_. Upon subsequent saccharification of dephenoliphied substrate, the hydrolysis yield was recorded as 46.88%. Optimized conditions such as using 1%+5% concentration of NH_3_+ H_3_PO_4_, for 30 min at 110°C temperature reduced total phenolic content (TPC) to 48 mg GAE/g DW. This reduction in phenolic content helped cellulases to act more proficiently on the substrate and saccharification yield of 55.06% was obtained. The findings will result in less utilization of cellulases to get increased yield of saccharides by hydrolyzing wheat straw, thus, making the process economical. Furthermore, pilot scale investigations of current study will help in upgrading the novel process to industrial scale.

## Introduction

In the present era, world is facing an inevitable energy crisis due to the depletion of fossil fuel deposits. With ever increasing population, the need to look for alternative energy resources has been a priority for most of the scientists around the world (Ramos et al., 2019). Recent studies indicate that the most promising alternative for non-renewable energy is the use of biofuels. For this purpose, lignocellulosic biomass which is mainly an agricultural waste, is mostly preferred (Novakovic et al., 2020). Lignin, cellulose, and hemicellulose are the main components of lignocellulosic biomass (Hasegawa et al., 2013). Among several agricultural wastes, wheat straw is considered as one of the most promising and abundant agricultural residues in the world (Ondrejoviè et al., 2020). According to Celignis analytical located in Ireland, the average yield of wheat straw is 1.3–1.4 kg/kg of wheat grain. Wheat straw being low cost and cheap agricultural by-product, coupled with the high cellulosic proportion (30–50%), makes wheat straw most suitable substrate for the production of bioethanol (Qiu et al., 2018).

The use of wheat straw for bioethanol production involves four basic steps i.e., pretreatment, enzymatic saccharification, fermentation, and down streaming of the product. Pretreatment of the feedstock is the most difficult step in the production of bioethanol using lignocellulosic biomass (Lynd et al., 2008). The complex arrangement of cellulose and hemicellulose hinders the access of enzyme to act on them due to the presence of lignin (Tareen et al., 2020). A variety of methods for pretreatment have been reported which include biological, chemical, mechanical, and thermochemical processes (Xiong et al., 2019).

Despite its primary importance in the process of biofuel formation, the pre-treatment step has certain disadvantages as it may result in the formation of inhibitory compounds (Ahmed et al., 2019). The main inhibitors produced during pretreatment are aliphatic acids such as formic acid, acetic acid, and levulinic acid, derivatives of furan including 5-hydroxymethylfurfural (HMF) and furfural, and various phenolic compounds i.e., phenol, *p*-hydroxybenzoic acid, and vanillin (Qi et al., 2014). These components are toxic or inhibitory to the cellulases and fermenting organisms. Therefore, these compounds must be removed or neutralized before the process of saccharification (Jönsson et al., 2013).

Various biological, physical, and chemical methods have been used for detoxification of lignocellulosic hydrolysate (Horváth et al., 2005). Most employed chemical detoxification methods include acidic and alkaline treatments (Chandel et al., 2011). Among alkalis, sodium hydroxide, aqueous ammonia, and calcium hydroxide are commonly used. Dilute acids including phosphoric acid and sulfuric acid are mostly used acids for detoxification of phenolic content produced during lysis of lignocellulose biomass (Mansour et al., 2016). However, removal of phenolic compounds from pretreated lignocellulosic biomass before enzymatic hydrolysis is rarely reported (Nawaz et al., 2017).

Recently we have demonstrated that the removal of these phenolic compounds can significantly increase the saccharification rate (Haq et al., 2018). However, application of this process at commercial level needs studies at pilot scale which will provide a better understanding of the process. Therefore, we have evaluated the challenges encountered during the up scaling of a dephenolification and subsequent saccharification of pre-treated wheat straw. In the present study, we have selected a pilot scale detoxification process for maximum saccharification under optimized conditions.

## Materials and Methods

### Chemicals

All chemicals used in the present study were of analytical grade and purchased from authentic suppliers of Sigma and Merck Ltd.

### Biomass

Pre-treatment of wheat straw and estimation of lignocellulosic content was carried out according to our previous report by Haq et al. (2018). For pretreatment, 2.5% sodium hydroxide (NaOH) was used for 10 min at a steaming temperature of 200°C in specialized boiler. The mesh size of biomass used was 2 mm. Cellulose and hemicelluloses content of biomass was estimated after Huang et al. (2010) and Lignin content was calculated according to TAPPI standards (TAPPI Standard T236cm-85, 1993). The lignocellulosic content and total phenolic content (TPC) of raw, pretreated and detoxified biomass is presented in Table 1.

**TABLE 1:** Lignocellulosic and Phenolic content of raw, pretreated and detoxified wheat straw samples.

Sr. No.	Biomass	Relative Cellulose content (%)	Hemi-cellulose content (%)	Lignin content (%)	Total Phenolic Content (mg GAE/g DW)
1	Wheat straw (raw)	48 ± 0.024	25 ± 0.013	19 ± 0.01	39 ± 0.019
2	Pre-treated Wheat straw	60 ± 0.031	19.3 ± 0.02	13 ± 0.05	210 ± 0.01
3	Detoxified wheat straw	69.5 ± 0.034	15 ± 0.04	5.9 ± 0.02	66 ± 0.03

### Cellulases

Thermophilic cellulases were produced by submerged fermentation using *Escherichia coli* BL-21 strain which was genetically modified with plasmid pET 21(b)+ cloned having genes from *Thermotoga petrophila* for endo-1,4-β-glucanase (EC 3.2.1.4), exo-1,4-β-glucanase (EC 3.2.1.91), and β-1,4-glucosidase (EC 3.2.1.21). These thermophilic cellulases were employed for saccharification studies after analyzing their activity against synthetic substrates i.e., Carboxy Methyl Cellulose (CMC) for endoglucanase, pNPC for exoglucanase and pNPG for beta-glucosidase. Table 2 shows the properties of enzymes used.

**TABLE 2:** Properties of hyperthermophilic cellulases.

Enzyme	Source	Host organism	Optimum temperature (°C)	Enzyme activity (U/mg)
Endo-1,4-β glucanase	*Thermotoga petrophila*	*E. coli* BL-21	90	10
Exo-1,4-β-glucanase	*Thermotoga petrophila*	*E. coli* BL-21	90	15
β-1,4-glucosidase	*Thermotoga petrophila*	*E. coli* BL-21	90	11525

### Removal of Phenolic Compounds

Pre-treated substrate (1 kg) was treated with 5 L of 5% alkalis i.e., Ca(OH)_2_ and NH_3_; acids i.e., H_2_O_2_, H_2_SO_4_, H_3_PO_4_; combinations (sequential addition) of NH_3_+H_3_PO_4_ and H_3_PO_4_+H_2_O_2_ at different temperatures i.e., 80, 90, 100, 110, 120, 130, 140, and 150°C for incubation periods of 5, 10, 15, 20, 25, 30, 35, and 40 min. A locally manufactured double jacketed, stainless steel vessel with automated temperature, pH, and agitation controls having working volume capacity of 20 L was used for this purpose. Traditional one-factor-at-a-time approach for optimization was followed. Afterward, substrates were rinsed with distilled water thrice. For this purpose, 5 L of water was added in the biomass and stirred at 50 rpm for 10 min, after which the water was removed. The same process was repeated two times for thorough washing. The substrates were then allowed to dry under room temperature before estimating TPC.

### Detection of Phenolic Content in Biomass

Folin–Ciocalteu (Folin and Ciocalteu, 1927) assay was used for the detection and estimation of TPC in wheat straw samples, before and after the detoxification of biomass. Assay was performed by adding 10 mg sample in a capped test tube with 9 ml distilled water and 1 ml Folin-Ciocalteu reagent. The contents were mixed vigorously and incubated for 5 min before addition of 10 ml 7% Na_2_CO_3_ solution. Subsequently, test tubes were kept at room temperature for 90 min. The optical density of the sample was measured at 550 nm (John et al., 2014). TPCs present in the biomass were estimated using the standard curve of Gallic acid (Nawaz et al., 2017).

### Enzymatic Saccharification

Enzymatic saccharification of detoxified biomass samples was initially carried out at laboratory scale using shake flask. Different parameters for the process were optimized by changing one factor at a time and keeping all the other factors constant. These parameters included temperature (50, 60, 70, 80, and 90°C), pH(5, 6, 7, 8, and 9), reaction time (0.5, 1, 2, 3, 4, and 5 h), and inoculum size (0.5, 1, 1.5, 2, 2.5, and 3%).

After optimizing the conditions at laboratory scale, detoxified wheat straw samples were analyzed for saccharification potential in a locally fabricated double jacketed stainless steel vessel (pilot scale). This vessel was equipped with heater, compressor, agitator, and digital controls for continuous monitoring of temperature and agitation change. Substrate (2.5% w/v) was added in 20 L of phosphate buffer (pH 7) along with 500 U of each of the three cellulase enzyme. The reaction was carried out at 80°C for a period of 3 h. Samples were drawn at regular intervals of 30 min to estimate total reducing sugar and TPC. Saccharification (%) was calculated using the following formula:

$$\%\mathrm{Saccharification}=\frac{R.S.\times \text{V}\times \mathrm{F1}}{\mathrm{F2}}\times 100$$

Where:

R.S. = Sugar concentration in hydrolysate estimated as total reducing sugar (mg/ml).

V = Total volume of the reaction mixture (ml).

F1 = Factor used for the conversion of monosaccharide to polysaccharide due to water uptake during hydrolysis (0.9 for hexoses).

F2 = Factor for carbohydrate content of substrate (total carbohydrate, mg/total substrate, mg).

### Scanning Electron Microscopy of Biomasses

Samples of wheat straw displaying best saccharification results after detoxification were sent to Center for Advance Studies in Physics (CASP), Government College University Lahore, Pakistan for Scanning Electron Microscopy (SEM). Sample preparation was not required as the samples were polymeric in nature.

### Statistical Analysis

The computer statistical software (SPSS 16.0) was used for the statistical analysis of the results. Significant difference among the replicates has been presented as Duncan’s multiple range tests in the form of probability (p) values (Duncan, 1995).

## Results and Discussion

### Choice of Detoxification Method

**FIGURE 1:**
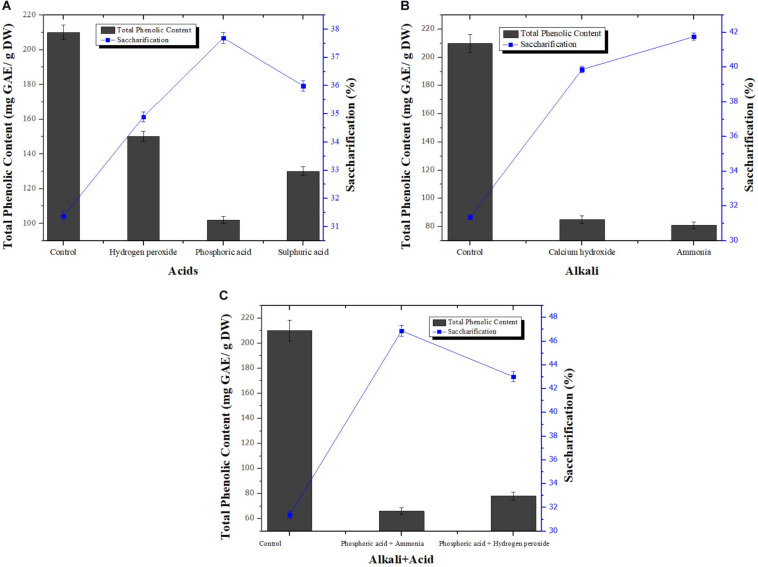
Effect of different acids **(A)**, alkalis **(B)**, and combination of alkali+acid **(C)** on dephenolification and saccharification of pretreated wheat straw. Y-error bars represent the standard deviation (SD ≤ ± 0.05) between three replicates.

Removal of phenolics from pretreated wheat straw using different alkalis [Ca(OH)_2_ and NH_3_], acids (H_2_O_2_, H_2_SO_4_, and H_3_PO_4_), combination of acids (H_2_O_2_+H_3_PO_4_), combination of acid and alkali (H_3_PO_4_+ NH_3_) was assessed. Optimization technique used was traditional one-factor-at-a-time. Subsequently, effect of phenolics removal from pretreated wheat straw on its enzymatic hydrolysis was also chronicled. TPC in control sample was found to be 210 ± 0.02 mg Gallic Acid Equivalent/gram of Dry Weight (GAE/g DW). Among all three acids used, phosphoric acid treated biomass showed maximum reduced phenolic content i.e., 102 ± 0.12 mg GAE/g DW as shown in Figure 1A. On the other hand, among alkalis, best results were obtained by aqueous ammonia that reduced TPC to 105 ± 0.04 mg GAE/g DW (Figure 1B). Combination of acids resulted in the removal of phenolic compounds to 78 mg GAE/g DW as evident from Figure 1C. Among all treatment strategies, combination of acid and alkali showed most efficient removal of phenolic content with total reduced phenolic content of 66 ± 0.02 mg GAE/g DW. All the pretreated samples of wheat straw processed for removal of phenolic content showed better saccharifcation i.e., 39.87 % [Ca(OH)_2_ treated sample], 36.01 % (NaOH treated sample), 41.76 % (NH_3_ treated sample) 34.89 % (H_2_O_2_ treated sample), 37.69 % (H_3_PO_4_ treated sample), 35.98 % (H_2_SO_4_ treated sample), 43.01 % (H_2_O_2_+H_3_PO_4_ treated sample), and 46.88 % (H_3_PO_4_+ NH_3_ treated sample) as compared to control sample (31.37 %) not treated for phenolics removal (Figure 1). Hence, it was considered that phosphoric acid used in combination with aqueous ammonia was the best method for detoxification of biomass. The removal of phenolic compounds is based on the fact that the treatment of biomass with phosphoric acid removes lignin that is present in biomass thus essentially removing phenolic contents. Moreover, cellulosic portion of the biomass remain unaffected by treatment with acid rather it provides more surface area for catalytic action of cellulases (Kim et al., 2011).

Treatment of biomass with ammonia may results in increase in internal surface area of cellulose which in turn decrease the degree of polymerization. Decrease in crystallinity or polymerization mediates the disruption of lignin content present in biomass that leads to the removal of phenolic inhibitors present in lignin portion along with lignin (Chen et al., 2020). Moreover, high cost of other alkalis hinders their application for detoxification while ammonia is volatile in nature so it is recyclable. Ammonia can be regenerated and reused again in the process of dephenoliphication (Kim and Holtzapple, 2006). By employing phosphoric acid together with aqueous ammonia, best result in terms of phenolic compounds removal was obtained because efficient fractionation of lignin and hemicellulose to remove phenolic derivatives is hard to achieve using dilute acid or alkali alone. Khobragade et al. (2004) found that removal of phenolic and other inhibitors increases under acidic conditions but detoxification further increased when alkaline conditions were provided along with acidic treatment. Wang et al. (2014) and Qiu et al. (2017) also used ammonia in combination with phosphoric acid for detoxification of wheat straw and rice straw respectively, and obtained results comparable to our findings.

### Effect of Incubation Time on Dephenoliphication

**FIGURE 2:**
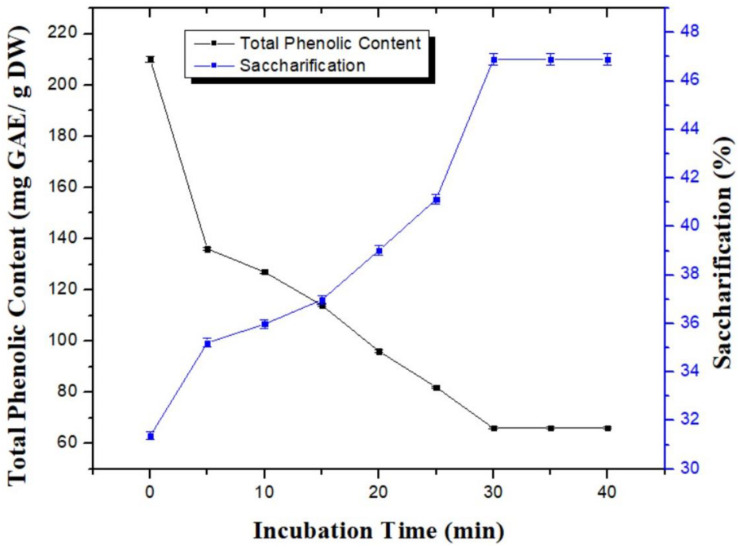
Effect of incubation time on the removal of phenlolics from pretreated wheat straw using combination of aqueous ammonia and phosphoric acid. Y-error bars represent the standard deviation (SD ≤ ± 0.05) between three replicates.

Detoxification of pretreated wheat straw using a combination of H_3_PO_4_ and NH_3_ was analyzed for variable time period i.e., 5, 10, 15, 20, 25, 30, 35, and 40 min to determine the optimum time period for maximum removal of TPC. In parallel, saccharification potential was also analyzed for substrate sample treated at various time periods to reduce phenolic content. Increase in incubation time resulted in gradual decrease of phenolic content. Maximum reduction in TPC was observed after 30 min of incubation (66 ± 0.06 mg GAE/g DW). Increase in incubation time beyond 30 min did not further decrease the TPC as shown in Figure 2. Similar pattern was noticed for the saccharification studies. Maximum saccharification value of 46.88; *p* < 0.05% was recorded using the substrate incubated for 30 min for dephenoliphication (Figure 2). Optimization of incubation time for the process is very important as shorter duration of time may not be able to remove sufficient amount of the phenolic compounds and longer time period could possibility lead to the formation of new phenolic compounds due to the fragmentation of soluble aromatic oligomers (Nilvebrant et al., 2003).

### Significance of Temperature on Dephenoliphication

**FIGURE 3:**
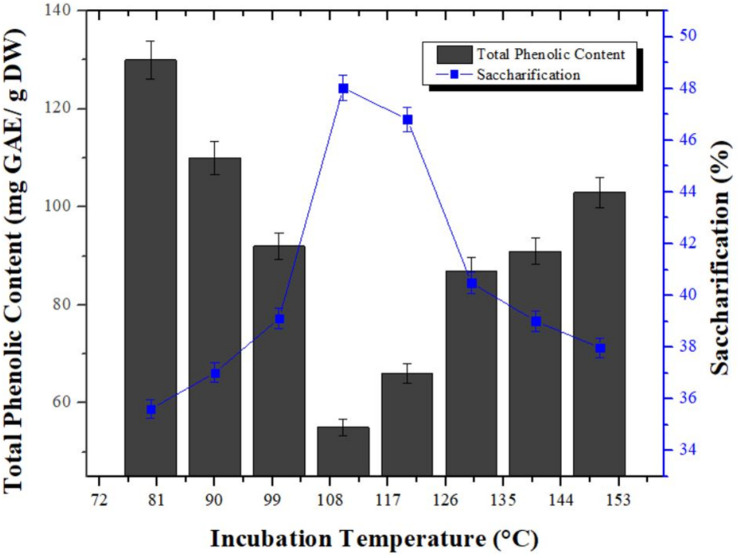
Studies of temperature influence on reduction in phenolic content from pretreated wheat straw. Y-error bars represent the standard deviation (SD ≤ ± 0.05) between three replicates.

Significance of temperature was assessed using different temperature range (80, 90, 100, 110, 120, 130, 140, and 150°C) for reduction in phenolic content of pretreated wheat straw obtained by combination of aqueous ammonia and phosphoric acid. In addition, saccharification study of dephenoliphied substrates at different temperature was also carried out. TPC was started to decrease with increase of temperature from 80°C (130 mg GAE/g DW) and maximum reduction in TPC (50 mg GAE/g DW) was observed at 110°C. However, further increase in the temperature ensued gradual increase in the TPC which was maximum (103 mg GAE/g DW) at 150°C. Analogous tendency was observed for enzymatic hydrolysis of dephenoliphied substrates and maximum saccharification i.e., 48.02% was determined for the substrate having least phenolic content. Substrate samples with increased phenolic content showed decreased saccharification (Figure 3). This may be due to the fact that with the increase in temperature, the hemicellulosic content is converted in to furans which can interfere with saccharification. The increase in TPC with time at higher temperature could be due the breakdown of ester bonds in lignin carbohydrate complexes and hence producing more phenolics (Canilha et al., 2008). Nawaz et al. (2017) reported maximum removal of phenolic compounds from pretreated sugarcane bagasse at 75°C after 120 min of incubation using Ca(OH)_2_ with 2.21 folds increase in saccharification. The conditions optimized are mild than used in current study might be due to difference in substrate used. However, no ample data is available on phenolic compounds removal from solid biomass for comparison.

### Optimal Concentration of Alkali and Acid Combination

**FIGURE 4:**
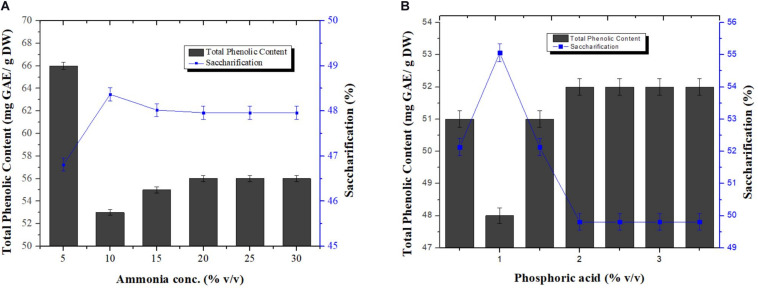
Effect of various concentrations of ammonia keeping the concentration of phosphoric acid constant (0.5%) **(A)**, and phosphoric acid keeping the concentration of ammonia constant (10%) **(B)**, on the reduction in phenolic content and saccharification of pretreated wheat straw. Y-error bars represent the standard deviation (SD ≤ ± 0.05) between three replicates.

Different concentrations of ammonia [5, 10, 15, 20, 25, and 30 % (v/v)] and phosphoric acid [0.5, 1, 1.5, 2, 2.5, 3, and 3.5% (v/v)] were investigated, in a one constant one variable manner, to find out the best concentrations for the combination in order to achieve maximum removal of phenolics from pretreated wheat straw. All samples treated for phenolics removal were also subjected to saccharification. Among all concentrations used, maximum reduction in phenolic content was observed at 5% (v/v) ammonia i.e., 53.12 mg GAE/g DW and 1% (v/v) phosphoric acid i.e., 48 mg GAE/g DW as evident from Figures 4A,B, respectively. Biomass treated at these concentrations for TPC reduction also showed maximum saccharification with a values of 48.37 and 55.06%, respectively. Low dose of acid and base maximally removed the phenolic content. No previous reports are available for utilization of ammonia and phosphoric acid in combination for removal of phenolic compounds from wheat straw.

### Scanning Electron Microscopy of Detoxified Wheat Straw

**FIGURE 5:**
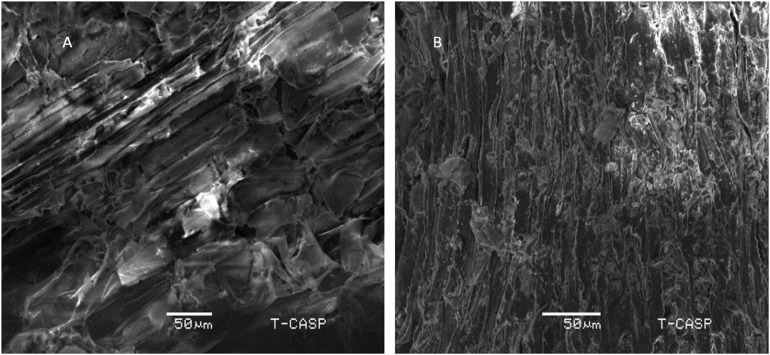
Scanning Electron Micrographs of **(A)** Pretreated wheat straw sample before phenolic reduction, ×50 **(B)** Pretreated wheat straw after reduction in total phenolic content.

Scanning electron micrographs of pretreated wheat straw samples, before and after detoxification under optimum conditions are shown in Figure 5. Before detoxification, the sample is in compact form, showing crystalline structure. On the other hand, detoxified sample shows loosely bound fibers and less crystallinity. The higher saccharification yield could be attributed to the structural changes in biomass thus making cellulose more assessable. However, evident from the previous study of Rajput et al. (2018), the heat treatment above 180 °C increases its digestibility of wheat straw due to degradation of hemicellulose. Hemicellulose degradation due to heating biomass at higher temperature (170 °C) has also been reported by Santucci et al. (2015). Since, the detoxification in present study is carried out at 110 °C, much less than 180 °C, it could be inferred that the higher saccharification reported is mainly the result of decreased TPC instead of simultaneous removal of hemicellulose due to detoxification conditions. Although changes in cellulose crystallinity and porosity cannot be ruled out, lignin and phenolic content can be considered here as the main discriminating parameter. However, the exact nature of structural changes is need to be studied in detail.

## Conclusion

It was concluded from current study that pilot scale removal of TPC from solid biomass has a significant effect on improved action of cellulases on pretreated wheat straw. Furthermore, treatment strategies and optimization parameters were found to have appreciable impact on removal of phenolic compounds. As the pilot scale studies related to current research are not previously available, there is a strong need to explore different strategies and different biomass for removal of phenolics and their assessment as a potential substrate for proficient conversion to fermentable saccharides enzymatically at industrial scale.

## Data Availability Statement

The raw data supporting the conclusions of this article will be made available by the authors, without undue reservation.

## Author Contributions

IH, KJ, and YX developed the idea and methodology for the study and helped in manuscript preparation. AN, BL, and YA performed the experiment and prepared the manuscript. XZ, FA, XF, and MS analyzed the results and interpreted them. All the authors proofread the manuscript.

## Conflict of Interest

The authors declare that the research was conducted in the absence of any commercial or financial relationships that could be construed as a potential conflict of interest.
